# Downregulation of METTL14 improves postmenopausal osteoporosis via IGF2BP1 dependent posttranscriptional silencing of SMAD1

**DOI:** 10.1038/s41419-022-05362-y

**Published:** 2022-11-01

**Authors:** Chaoqing Huang, Yuan Wang

**Affiliations:** Department of Orthopedics, Second Affiliated Hospital of Navy Medical University, Shanghai, 200003 China

**Keywords:** Cell biology, Cancer

## Abstract

Osteoporosis (OP) tends to occur in postmenopausal women, making them prone to fractures. *N*6-methyladenosine (m6A) methylation plays a crucial role in OP. Herein, we aimed to explore the effects of METTL14 on osteogenesis and the underlying mechanism. Osteogenic differentiation was assessed through osteoblast markers expression, cell proliferation, ALP activity, and mineralization, which were detected by qRT-PCR, CCK-8, EdU assay, ALP staining assay, and ARS staining assay, respectively. Osteoporosis was evaluated in OVX mice using qRT-PCR, microcomputed tomography, and H&E staining assay. The levels of METTL14 and SMAD1 were measured using qRT-PCR and western blot, and their interaction was assessed using RIP and luciferase reporter assay. M6A methylation was analyzed using the Me-RIP assay. The results indicated that m6A, METTL14, and SMAD1 levels were downregulated in patients with OP and OVX mice, and upregulated in osteogenic BMSCs. Knockdown of METTL14 suppressed osteogenesis of BMSCs and reduced bone mass of OVX mice. Moreover, silencing of METTL14 positively related to SMAD1 and inhibited m6A modification of SMAD1 by suppressing its stability. IGF2BP1 was identified as the methylation reader, and which knockdown reversed the upregulation induced by SMAD1. Overexpression of SMAD1 reversed the suppression of osteogenic differentiation induced by METTL14 knockdown. In conclusion, interference with METTL14 inhibited osteogenic differentiation of BSMCs by m6A modification of SMAD1 in an IGFBP1 manner, suggesting that METTL14 might be a novel approach for improving osteoporosis.

## Introduction

Osteoporosis (OP) is a systemic bone disease, characterized by increased bone fragility and fracture risk. OP is commonly caused in older adults, especially in postmenopausal women. Estrogen is important in the pathogenesis of OP, and postmenopausal women’s ovaries stop secreting estrogen, which is more likely to cause OP [1]. OP is asymptomatic in its early stages and often goes undiagnosed until it presents as a low-trauma fracture [2]. Even if diagnosed with OP, the management and prevention of fractures are often underappreciated by clinicians and patients [3]. With the understanding of the pathogenesis of OP, the imbalance of bone remodeling is the main cause of OP, including bone resorption and formation processes [4]. Bone remodeling occurs in bone trabeculae and cortical bone. More bone remodeling units and remodeling imbalances cause accelerated bone loss and cause OP in postmenopausal women [5]. Thus, treatment with bisphosphonates (bone resorptive inhibitors) and teriparatide (bone formation stimulants) contributes to improving OP and reducing fracture risk [6]. At present, stem cell therapy provides a new approach to treating various diseases. Bone marrow-derived mesenchymal stem cells (BSMCs) have a positive role in promoting osteogenesis, and their efficacy in treating OP has been demonstrated in preclinical animal models [7]. However, how to induce or promote the osteogenic differentiation of BSMCs needs more in-depth research.

Methylation modification is one of the main forms of RNA modification, among which N6-methyladenosine (m6A) methylation is a widespread methylation modification in eukaryotic RNA [8]. M6A is installed by m6A methyltransferases (writers), removed by m6A demethylases (erasers), and recognized by reader proteins (readers), participating in the regulation of RNA metabolisms such as translation, splicing, processing, and degradation [9, 10]. M6A methylation modification presents in most RNAs and regulates a variety of cellular biological processes, including immune regulation, metabolism, stem cell maintenance, and body development [11]. Once m6A is disturbed, various diseases will occur. METTL14, an important m6A methyltransferase, regulates the fate of stem cells [11]. A previous study showed that METTL14-based m6A modification enhances osteoblast activity and promotes bone formation [12]. However, even though METTL14 is essential for m6A modification, the biological functions of METTL14 and underlying mechanisms remain to be fully elucidated.

In this study, we sought to clarify the effect of METTL14 on the osteogenesis of BSMCs and bone mass in mice after ovariectomy. We also investigate the mechanism of osteogenic differentiation, which is linked to METTL14-dependent m6A modification of SMAD1. This study will provide a new way to improve postmenopausal OP.

## Materials and methods

### Clinical samples

The clinical protocol was approved by the Ethics Committee of the Second Affiliated Hospital of Navy Medical University. Sixty postmenopausal women were enrolled in this study, containing patients with OP (*n* = 30) and patients with other bone diseases instead of OP (*n* = 30). All subjects with other diseases and smoking or drinking histories were excluded. Bone samples were collected from all subjects. All participants signed written informed consent.

### Cell culture

Human BMSCs were purchased from ATCC (Manassas). All cells were maintained in BMSC complete medium (Procell, Wuhan) at 37 °C with 5% CO_2_. BMSCs in passages 4–6 were used in the following experiments.

### Osteogenic differentiated cell model

An OriCell human BMSCs osteogenic induction kit was purchased from Cyagen (Guangzhou). Six-well plates were precoated with 0.1% gelatin. BMSCs were seeded into 6-well plates at the density of 2 × 10^4^ cells/cm^2^. When cell confluence reached 70%, 2 ml osteogenic-induced medium was added to incubate with BMSCs. The medium was replaced every 3 days. The differentiated BMSCs were harvested after 21 days.

### Ovariectomized (OVX) mouse model

All animal experiments were approved by the Ethics Committee of the Second Affiliated Hospital of Navy Medical University. Female C57BL/6 mice (8 weeks old) were randomly divided into four groups (five mice per group). The mice with bilateral ovaries removed after anesthesia were OVX mice. The mice with both ovaries exposed and adipose tissue removed were the sham group. The procedure for removing the ovaries was as follows. Mice were anesthetized, their backs were made with a 50 mm incision. Ovaries on both sides were exposed after removing the muscle tissues. Ovaries are removed after tubal ligation, then the wound was sutured. Eight weeks after OVX, all mice were euthanized.

Lentiviruses that overexpress METTL14 (Lv-METTL14) and corresponding negative control (Lv-nc) were synthesized by Genepharma (Shanghai) and intramuscularly vertically injected into the lateral thigh muscle of the mice. Three consecutive injections were performed every 2 days. After injecting for 7 days, mice received bilateral ovaries removal. Eight weeks after OVX, all mice were euthanized.

### Determination of osteoporosis-related indicators

After the mice were euthanized, their right femurs were dissected. Then the femurs were fixed with 4% paraformaldehyde (Sigma-Aldrich, St. Louis) for 24 h. The microstructure of bone was analyzed by microcomputed tomography (micro‐CT) system (GE Healthcare, Little Chalfont). We analyzed trabecular bone volume per tissue volume (BV/TV), trabecular number (Tb.N), trabecular thickness (Tb.th), and trabecular separation (Tb.Sp). Additionally, bone mineral density (BMD) was detected using an X-ray bone density analyzer (Bioemtech, Shanghai).

### Detection of m6A level

M6A level was evaluated using the EpiQuik m6A RNA methylation quantification kit (Epigentek, Farmingdale). Briefly, after isolating total RNA, RNA was incubated with 2 μl NC or m6A positive control at 37 °C for 90 min. Then, m6A RNA was captured using a capture antibody and detection antibody. Following adding 100 μL developer for 10 min, stop the reaction. OD value was read using a microplate reader (DiaTek, Wuxi) at 450 nm.

### Cell transfection

short hairpin RNA (Sh)-METTL14, sh-YTHDF1, sh-YTHDF2, sh-YTHDC1, sh-IGF2BP1, sh-IGF2BP2, sh-IGF2BP3, sh-NC, empty vector, and SMAD1 overexpression vector, IGF2BP1 overexpression vector, and IGF2BP2 overexpression vector were obtained from Genepharma. BMSCs were seeded into 6-well plates and transfected with sh-METTL14 and sh-NC using Lipofectamine 3000 (Invitrogen, Carlsbad) for 48 h.

### qRT-PCR

Total RNA was isolated from right femurs and BMSCs using a total RNA isolation kit (Beibei Biotechnology, Zhenzhou). After testing RNA concentration and integrity, the cDNA first chain was synthesized using a reverse transcriptase kit (Beibei Biotechnology). Then, qPCR was conducted using real-time PCR (SYBR Green) Mixture (Beibei Biotechnology). The reactions were performed on Rotor-Gene 3000 system (Corbett, Sydney) with the conditions: 95 °C for 1 min, 40 cycles of 95 °C for 5 s, and 60 °C for 15 s. The mRNA expression (folds) was calculated using the 2^−ΔΔCt^ method. GAPDH was the normalization.

### H&E staining assay

All mice femurs were immobilized with 4% paraformaldehyde for 24 h and decalcified in EDTA for 20 days. Then the femurs were embedded in paraffin and cut into paraffin sections. Sections were dewaxed with xylene and rehydrated with gradient ethanol. After staining using hematoxylin for 4 min, the sections were continued to stain with eosin for 1.5 min. Then the sections were dehydrated using ethanol and treated with xylene. The sections were visualized under a microscope (Olympus, Tokyo). The bone surface (BS) was quantified.

### Western blot

BMSCs were lysed using RIPA buffer (Sigma-Aldrich). Following detecting protein concentration, cell lysate was isolated using 10% SDS-PAGE, followed by being transferred on PVDF membranes. After incubating with primary antibodies (anti-BGLAP, anti-COL1A1, anti-RUNX2, anti-OCN, anti-OPN, anti-METTL14, anti-FTO, anti-ALKBH5, anti-WTAP, anti-RBM15, anti-VIRMA, anti-METTL3, anti-SMAD1, and anti-GAPDH, Abcam, Cambridge) at 4 °C overnight, the membranes were incubated with HRP-conjugated secondary antibody (Abcam) at 25 °C for 1 h. Bands were visualized using immobilon ECL ultra western HRP substrate (Sigma-Aldrich). The original bands are shown in the Supplementary material.

### CCK-8 analysis

BMSCs seeded into 96-well plates were incubated at 37 °C for 0, 1, 2, and 3 days. Afterward, 10 μL CCK-8 (Dojindo Kumamoto) was incubated with cells for another 4 h. OD value was evaluated using a microplate reader at 450 nm.

### EdU analysis

Cell proliferation was analyzed using an EDU kit (Ribobio). BMSCs seeded into 96-well plates were incubated with 100 μL EDU for 2 h. Then, the cells were fixed with 4% paraformaldehyde for 15 min, incubated with 0.2% glycine for 10 min, and permeabilized with 0.5% Triton-X100 for 10 min. Apollo staining solution was incubated with cells in the dark for 30 min, and DAPI was added to incubate for 10 min. The stained cells were imaged using a fluorescence microscope (Olympus).

### ALP assay

BMSCs were immobilized with 4% paraformaldehyde for 20 min. After washing with PBS, the cells were incubated with 1 mL ALP staining solution at 37 °C for 1 h, The results were imaged under a microscope (Olympus). The ALP staining intensity was quantified using the Image J software (National Institutes of Health).

### ARS staining assay

After osteogenic induction for 3 weeks, BMSCs were fixed with 2 mL 4% paraformaldehyde for 0.5 h. After washing with PBS, 2 mL ARS solution was stained with BMSCs for 10 min. PBS was used to wash the stain solution. The stained cells were visualized under a microscope (Olympus). The ARS staining intensity was quantified using the Image J software.

### Me-RIP assay

Me-RIP was performed using the Me-RIP m6A transcriptome profiling kit (Ribobio, Guangzhou). Briefly, total RNA was fragmented by incubating with the RNA fragmentation buffer. The Me-RIP reaction solution was prepared by adding 395 μL fragmented RNA, 5 μL RNase inhibitor, and 100 μL IP buffer. Magnetic beads A/G were incubated with IP buffer and 5 μg anti-m6A antibody. The Me-RIP reaction solution was incubated with anti-m6A magnetic beads at 37 °C for 2 h. After the beads were eluted, RNA was purified and recycled. The m6A level of SMAD1, Smad5, and Smad8 was detected using qPCR.

### Bioinformatics

The m6A methylation sites of SMAD1 were predicted using the SRAMP database (http://www.cuilab.cn/sramp). The interaction of SMAD1 and other factors in SMAD1 signaling was predicted using the STRING online tool (https://cn.string-db.org/).

### RNA immunoprecipitation (RIP) assay

RIP kit was acquired from GeneSeed (Guangzhou, China). BSMCs were lysed using 1 ml buffer A and centrifugation at 14,000×*g* for 10 min. Protein A + G beads were incubated with anti-METTL14 and anti-IgG (negative control) at 4 °C for 2 h. The lysate was incubated with beads at 4 °C overnight. After washing five times, RNA was isolated and purified. The enrichment of SMAD1 was detected by qRT-PCR.

### Luciferase reporter assay

The wild-type (WT) sequences of SMAD1 containing site 1 or site 2 were inserted into the Pmir-GLO vector (Promega, Madison). The corresponding mutant sequences of SMAD1 containing site 1 or site 2 were also cloned into the Pmir-GLO vector. BSMCs seeded into 24-well plates were co-transfected with WT or MUT plasmids and sh-nc or sh-METTL14 using Lipofectamine 3000. On the other hand, BSMCs were co-transfected with WT or MUT plasmids and vector or METTL14/IGF2BP1/IGF2BP2 overexpression vector using Lipofectamine 3000. Moreover, BSMCs were co-transfected with WT or MUT plasmids and sh-nc or sh- IGF2BP1/sh-IGF2BP2 using Lipofectamine 3000. After 48 h, the luciferase activity was detected using the dual-luciferase reporter assay system (Promega).

### Detection of SMAD1 stability

BSMCs were treated with 2 µg/mL actinomycin D and incubated for 0, 1, 2, 4, and 8 h. Then, the expression of SMAD1 was evaluated using qRT-PCR. The SMAD1 protein stability was assessed using the cycloheximide (CHX) pulse-chase assay.

### Statistical analysis

All data were analyzed by GraphPad Prism 7.0 software and presented as mean ± SD. Differences were performed by student’s *t*-test and one-way ANOVA followed by Turkey post hoc test. *P* < 0.05 was considered statistically significant.

## Results

### M6A expression in patients with OP, osteogenic differentiated cells, and OVX mice

**Fig. 1:**
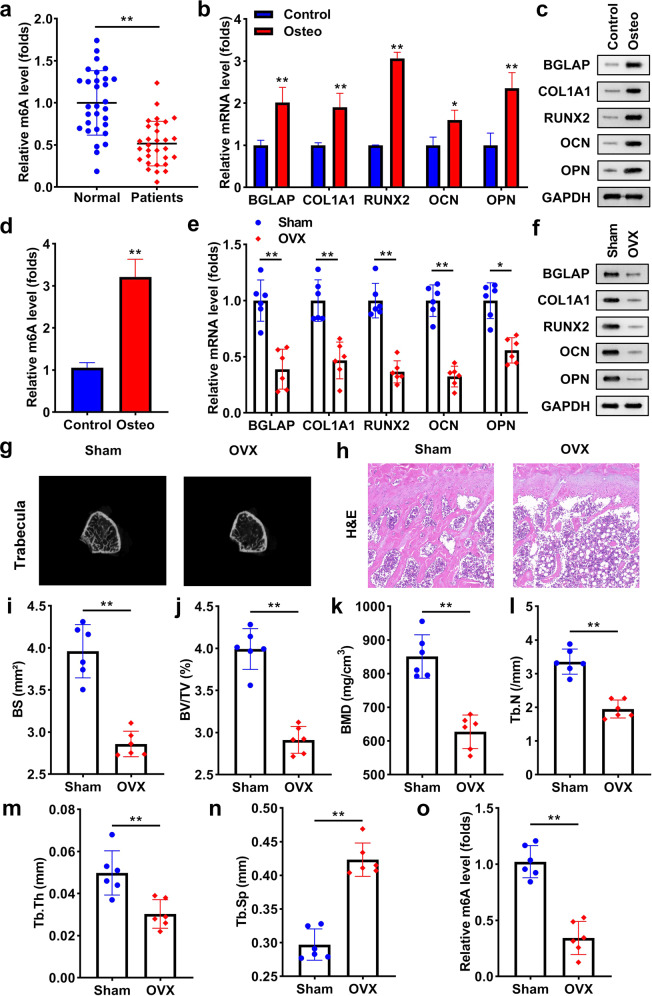
M6A expression in patients with OP, osteogenic differentiated cells, and OVX mice. **a** m6A level in patients with or without OP (both *n* = 30). **b** The levels of BGLAP, COL1A1, RUNX2, OCN, and OPN were detected using qRT-PCR. **c** The protein levels of BGLAP, COL1A1, RUNX2, OCN, and OPN in the differentiated and undifferentiated BMSCs. **d** m6A level in osteoblast differentiated or undifferentiated cells. **e** The mRNA levels of BGLAP, COL1A1, RUNX2, OCN, and OPN were detected in mice of the sham and OVX groups. **f** The protein levels of BGLAP, COL1A1, RUNX2, OCN, and OPN in the sham and OVX mice. **g** Images of trabecular bone of mice. **h** Bone formation in mice was measured using the H&E assay and **i** BS was quantified. Bone formation index containing **j** BV/TV, **k** BMD, **l** Tb.N, **m** Tb.th, and **n** Tb.Sp were analyzed by μ-CT. **o** m6A levels in mice were tested using qRT-PCR. **P < 0.01 and *P < 0.05.

To evaluate whether m6A occurs in OP, we found the level of m6A was significantly low expressed in the patients with OP, compared with normal individuals (Fig. 1a). Then, we established an osteoblast differentiation cell model. As indicated in Figs. 1b and 1c, osteogenic markers, including BGLAP, COL1A1, RUNX2, OCN, and OPN were markedly upregulated in the differentiated cells. In osteogenesis BMSCs, the m6A level was notably higher than in control cells (Fig. 1d). To establish an osteoporosis mice model, their ovaries were removed. BGLAP, COL1A1, RUNX2, OCN, and OPN mRNA and protein levels were markedly downregulated in OVX mice (Fig. 1e, f). According to the photographs of trabecular bone, the bone mass was notably lower in OVX mice than in sham mice (Fig. 1g). The results of the H&E staining assay showed that the bone surface (BS) and bone volume were reduced, and fat cells were increased in the OVX mice (Fig. 1h, i). Moreover, OVX significantly reduced BV/TV, BMD, Tb.N, Tb.th, and increased Tb.Sp (Fig. 1j-–n). The level of m6A was significantly downregulated in OVX mice, which was analyzed using qRT-PCR (Fig. 1o).

### METTL14 expression is reduced in osteoporosis

**Fig. 2:**
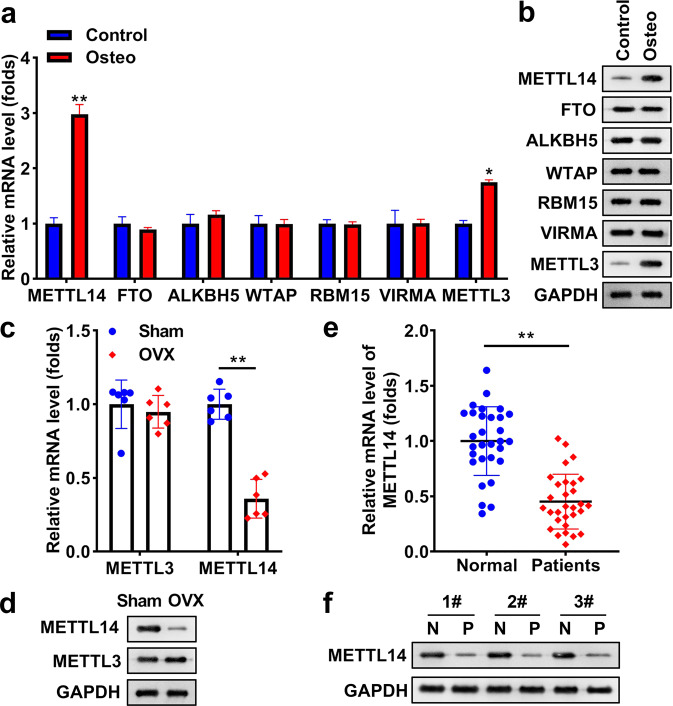
METTL14 is reduced in osteoporosis. **a** The levels of METTL14, FTO, ALKBH5, WTAP, RBM15, VIRMA, and METTL3 were examined in differentiated or undifferentiated BSMCs using qRT-PCR. **b** The protein levels of METTL14, FTO, ALKBH5, WTAP, RBM15, VIRMA, and METTL3 were examined in differentiated or undifferentiated BSMCs using a western blot. **c** METTL14 and METTL3 at mRNA expression was tested in sham mice and OVX mice. **d** The protein levels of METTL14 and METTL3 was measured in sham and OVX mice using western blot. **e** METTL14 in patients with or without OP (both *n* = 30). **f** The protein levels of METTL14 in normal and OP subjects. **P < 0.01 and *P < 0.05.

In osteogenic differentiated BMSCs, methylation “writer” METTL14 and METTL3 were significantly upregulated, especially METTL14. However, there was no significant difference in FTO, ALKBH5, WTAP, RBM15, and VIRMA between control and differentiated BMSCs (Fig. 2a, b). Thus, we only detected METTL14 and METTL3 expression in sham mice and OVX mice, and the data showed that only METTL14 was markedly decreased in OVX mice, but there was no significant difference in METTL3 between the two group mice (Fig. 2c, d). Finally, we focused on the levels of METTL14 in subjects and found that METTL14 was also reduced in patients with OP, compared with other subjects (Fig. 2e, f).

### Knockdown of METTL14 inhibits osteogenic differentiation

**Fig. 3:**
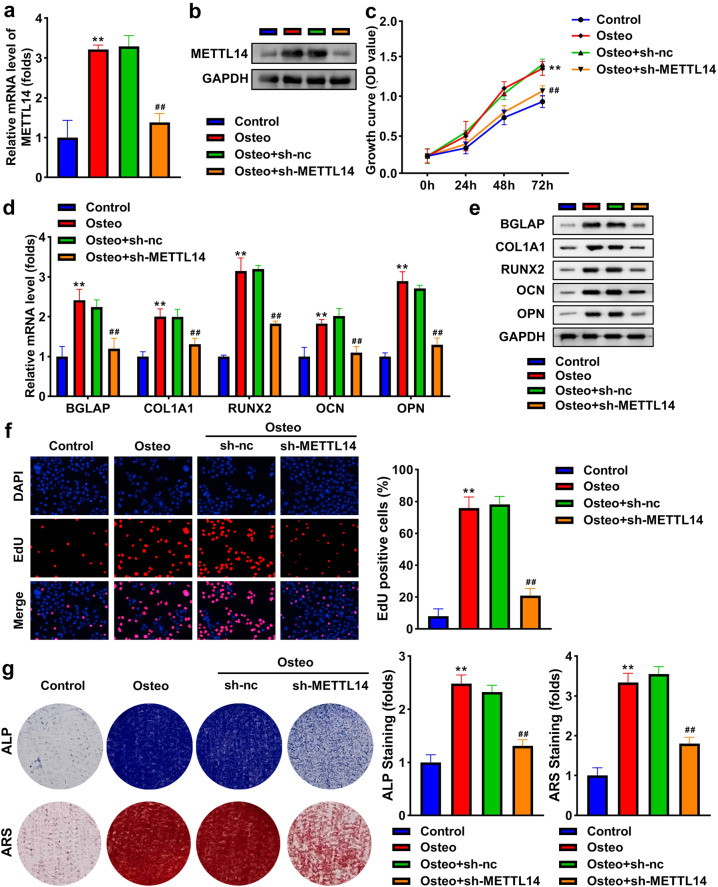
Knockdown of METTL14 inhibits osteogenic differentiation. METTL14 in osteogenic BMSCs following transfection was examined by **a** qRT-PCR and **b** western blot. **c** Cell growth was assessed using the CCK-8 assay. **d** The mRNA expression of BGLAP, COL1A1, RUNX2, OCN, and OPN. **e** The protein levels of BGLAP, COL1A1, RUNX2, OCN, and OPN. **f** Cell proliferation was assessed using EDU assay. **g** Osteogenesis was analyzed using ALP staining assay and ARS staining assay. The staining intensity was quantified. **P < 0.01 vs. control. ##*P* < 0.01 vs. osteo + sh-nc.

To study the role of METTL14 in BSMCs, we used qRT-PCR and western blot to detect its expression. The results indicated that METTL14 was significantly elevated in differentiated BSMCs, which were significantly reduced by transfection of sh-METTL14 (Fig. 3a, b). Knockdown of METTL14 decreased the expression of BGLAP, COL1A1, RUNX2, OCN, and OPN in osteogenic BSMCs (Fig. 3d, e). Cell growth and proliferation were significantly promoted in osteogenic BMSCs, but the knockdown of METTL14 suppressed cell growth and proliferation (Fig. 3c, f). Interference with METTL14 abolished the promotion of ALP activity and mineralization induced by osteogenic treatment (Fig. 3g).

### METTL14 improves osteoporosis of OVX mice

**Fig. 4:**
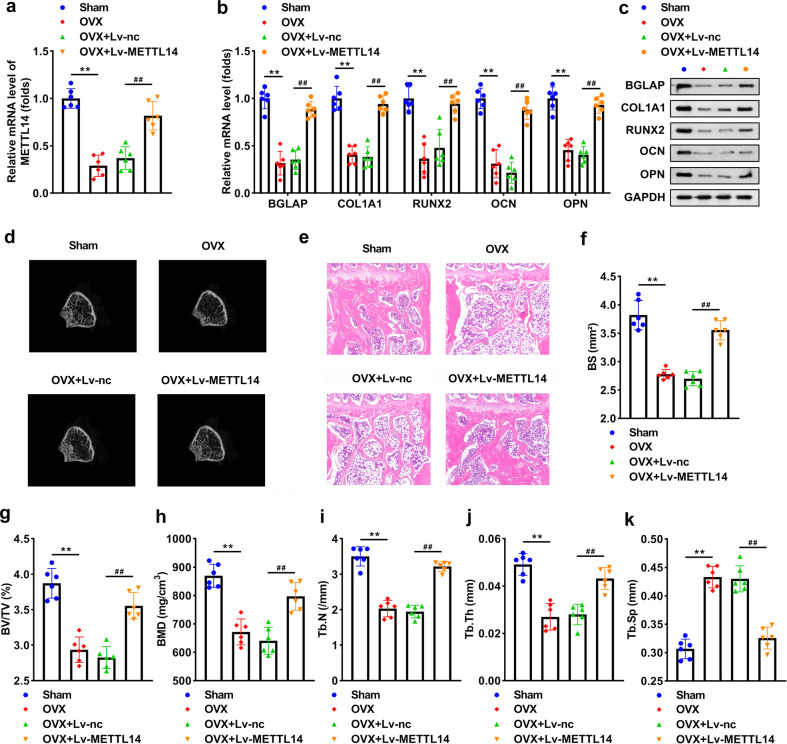
METTL14 improves osteoporosis of OVX mice. **a** METTL14 expression in OVX and lv-METTL14 transfected OVX mice. **b** The mRNA expression of BGLAP, COL1A1, RUNX2, OCN, and OPN. **c** The protein levels of BGLAP, COL1A1, RUNX2, OCN, and OPN. **d** Images of trabecular bone of mice. **e** Bone formation in mice was measured using the H&E assay and **f** BS was quantified. Bone formation index containing **g** BV/TV, **h** BMD, **i** Tb.N, **j** Tb.th, and **k** Tb.Sp were analyzed by μ-CT. **P < 0.01 vs. control. ##*P* < 0.01 vs. OVX + Lv-nc.

OVX induced a significant reduction of METTL14, and transfection with Lv-METTLE14 increased its level (Fig. 4a). Overexpression of METTL14 notably elevated BGLAP, COL1A1, RUNX2, OCN, and OPN levels in OVX mice (Fig. 4b, c). OVX induced bone mass loss, while overexpressing METTL14 abolished the loss (Fig. 4d). The results of the H&E staining assay revealed that the BS and bone volume were improved by METTL14 in OVX mice (Fig. 4e, f). Furthermore, METTL14 reversed the reduction of BV/TV, BMD, Tb.N, Tb.th, and the increase of Tb.Sp induced by OVX (Fig. 4g–k).

### Knockdown of METTL14 positively regulates SMAD1

**Fig. 5:**
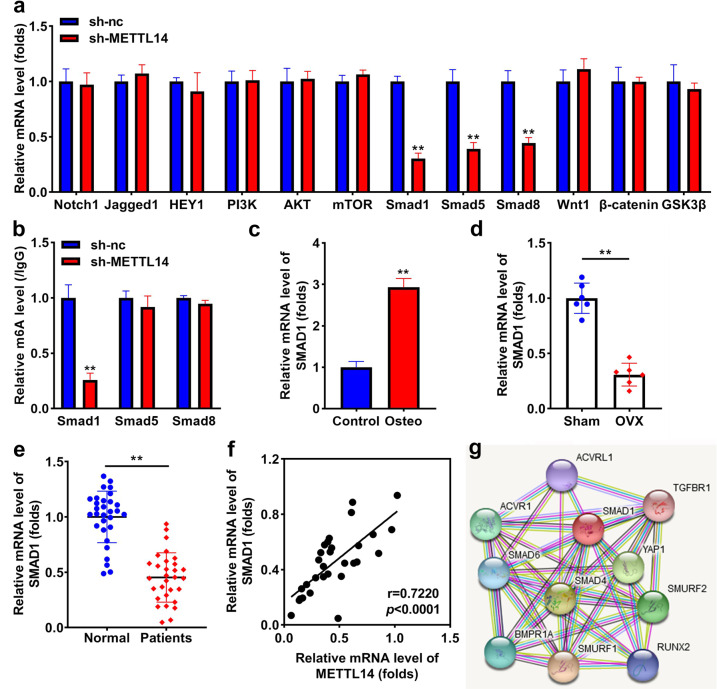
Knockdown of METTL14 positively regulates SMAD1. **a** The mRNA levels of Notch1, Jagged1, HEY1, PI3K, AKT, mTOR, SMAD1, Smad5, Smad8, Wnt1, β-catenin, and GSK3β after transfection with METTL14. **b** m6A modification of Smad1, Smad5, and Smad8 following sh-METTL14 transfection. **c** SMAD1 expression in osteogenic differentiated and undifferentiated BSMCs. **d** SMAD1 expression in sham and OVX mice. **e** SMAD1 expression in patients with or without OP (both *n* = 30). **f** The relationship between METTL14 and SMAD1 was assessed by Pearson correlation coefficient (**g**) SMAD1 related proteins were predicted using the STRING online tool. **P < 0.01.

To investigate the underlying mechanism of METTL14, we found that the knockdown of METTL14 significantly downregulated the expression of Smad1, Smad5, and Smad8, but did not influence the expression of Notch1, Jagged1, HEY1, PI3K, AKT, mTOR, Wnt1, β-catenin, and GSK3β (Fig. 5a). In addition, interference with METTL14 notably inhibited m6A methylation of SMAD1 rather than Smad5 and Smad8 (Fig. 5b). SMAD1 expression was markedly increased in osteogenic BMSCs (Fig. 5c), and markedly decreased in OVX mice (Fig. 5d). SMAD1 was notably downregulated in patients with OP (Fig. 5e), and its expression was positively related to METTL14 expression (*r* = 0.7220, *P* < 0.0001; Fig. 5f). SMAD1 regulating SMAD1 pathway-related factors were predicted (Fig. 5g).

### METTL14 promotes m6A methylation of SMAD1

**Fig. 6:**
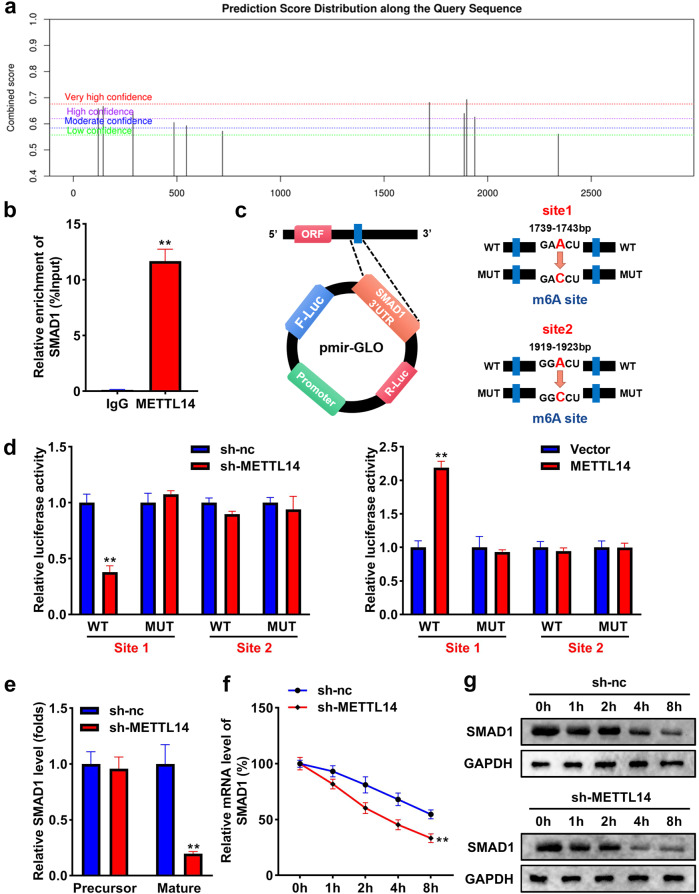
METTL14 promotes m6A methylation of SMAD1. **a** m6(A) methylation sites of SMAD1 were predicted using the SRAMP database. **b** The combination between METTL14 and SMAD1 was analyzed using RIP assay. **c** The potential bindings sites in SMAD1 were shown. **d** The luciferase reporter analysis evaluated which site of SMAD1 could be bound to METTL14. **e** Precursor and maturation of SMAD1 levels were assessed using qRT-PCR. **f** The stability of SMAD1 at the mRNA level. **g** The stability of SMAD1 at the protein level. **P < 0.01.

The bioinformatic analysis results showed that there are multiple methylation binding sites of SMAD1 (Fig. 6a). The results of the RIP assay demonstrated that METTL14 could bind to SMAD1 (Fig. 6b). Two potential binding sites were found (Fig. 6c). The data of the luciferase reporter assay indicated that knockdown of METTL14 significantly decreased the luciferase activity in the WT group with site 1 rather than site 2, whereas overexpression of METTL14 elevated the luciferase activity with site 1 instead of site 2 in the WT group (Fig. 6d). Subsequently, we explored the effect of METTL14 on the stability of SMAD1. As shown in Fig. 6e, the knockdown of METTL14 reduced mature SMAD1 expression instead of its precursor. Moreover, the stability of SMAD1 was significantly reduced by silencing METTL14 with the increase of time at both mRNA and protein levels (Fig. 6f, g).

### IGF2BP1 is positively related to SMAD1

**Fig. 7:**
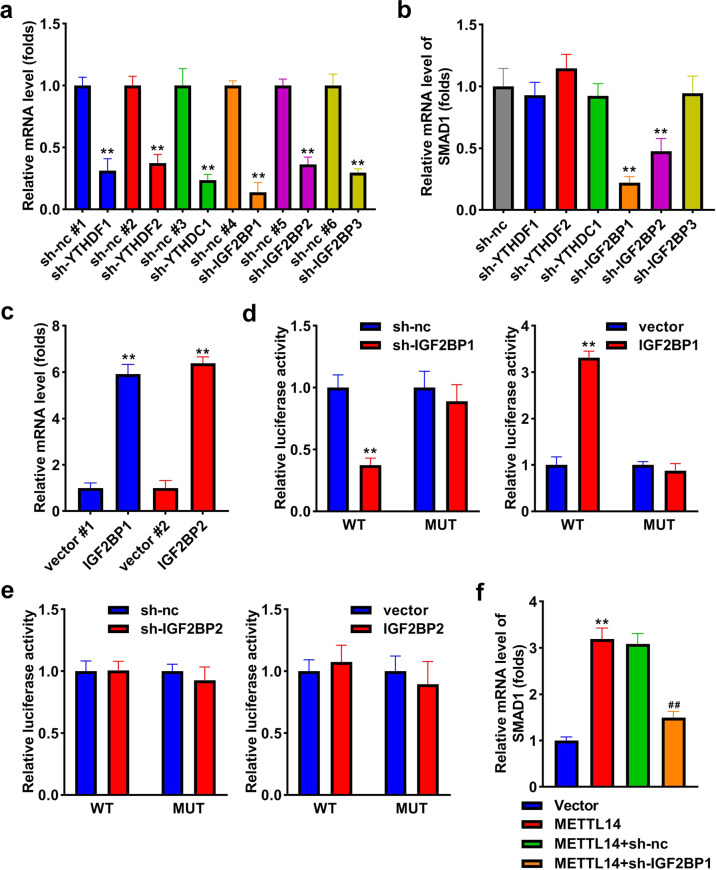
IGF2BP1 is positively related to SMAD1. **a** The levels of YTHDF1, YTHDF2, YTHDC1, IGF2BP1, IGF2BP2, and IGF2BP3 after transfection. **b** qRT-PCR measured SMAD1 after the knockdown of YTHDF1, YTHDF2, YTHDC1, IGF2BP1, IGF2BP2, and IGF2BP3. **c** IGF2BP1 and IGF2BP2 levels after transfection. **d** The relationship between IGF2BP1 and SMAD1 was assessed by luciferase reporter assay. **e** The relationship between IGF2BP2 and SMAD1 was assessed by luciferase reporter assay. **f** SMAD1 expression was measured after overexpression of METTL14 and knockdown of IGF2BP1. **P < 0.01. ##*P* < 0.01 vs. sh-METTL14 + vector.

To explore which m6A reader acts in osteogenic differentiation, we knockdown the levels of YTHDF1, YTHDF2, YTHDC1, IGF2BP1, IGF2BP2, and IGF2BP3, and their expression was all decreased after transfection (Fig. 7a). Then, SMAD1 was markedly downregulated by sh-IGF2BP1 and sh-IGF2BP2, especially sh-IGF2BP1 (Fig. 7b). To explore whether IGF2BP1 and IGF2BP2 could bind to SMAD1, we transfected their overexpressed vectors, and their levels were significantly upregulated (Fig. 7c). The results of luciferase reporter assay indicated that only IGF2BP1 could interact with SMAD1, while IGF2BP2 could not interact with SMAD1 (Fig. 7d, e). Moreover, overexpression of METTL14 increased the expression of SMAD1, whereas knockdown of IGF2BP1 reversed the upregulation of SMAD1 induced by METTL14 (Fig. 7f).

### Knockdown of METTL14 suppresses osteogenic differentiation by modulating SMAD1

**Fig. 8:**
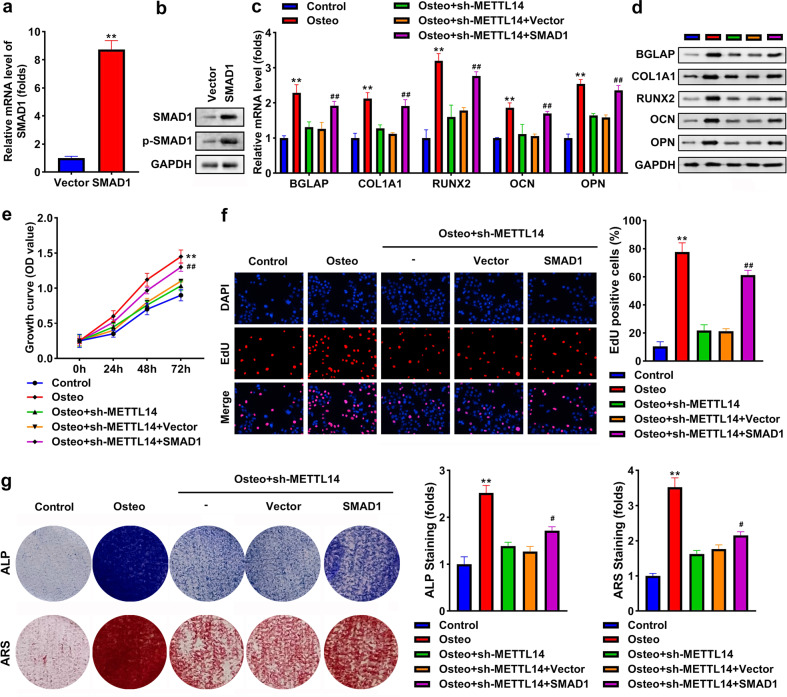
Knockdown of METTL14 suppresses osteogenic differentiation by modulating SMAD1. **a** SMAD1 expression at mRNA level post-transfection. **b** SMAD1 and p-SMAD1 protein levels post-transfection. **c** The mRNA expression of BGLAP, COL1A1, RUNX2, OCN, and OPN. **d** The protein levels of BGLAP, COL1A1, RUNX2, OCN, and OPN. **e** Cell growth was assessed using the CCK-8 assay. **f** Cell proliferation was assessed using EDU assay. **g** Osteogenesis was analyzed using an ALP staining assay and an ARS staining assay. The staining intensity was quantified. **P < 0.01 in (**a**) vs. vector. **P < 0.01 in (**b**–**g**) vs. control. ##*P* < 0.01 vs. osteo + sh-METTL14 + vector.

After transfection with the SMAD1 overexpression vector, SMAD1 at mRNA and protein levels, and p-SMAD1 levels were all elevated (Fig. 8a, b). Overexpression of SMAD1 reversed the downregulation of BGLAP, COL1A1, RUNX2, OCN, and OPN induced by the knockdown of METTL14 (Fig. 8c, d). Additionally, the knockdown of METTL14 suppressed cell growth and proliferation, whereas SMAD1 rescued the suppression (Fig. 8e, f). Moreover, ALP activity and mineralization of osteogenic cells were both inhibited by silencing of METTL14, which were abrogated by SMAD1 overexpressing (Fig. 8g).

## Discussion

**Fig. 9:**
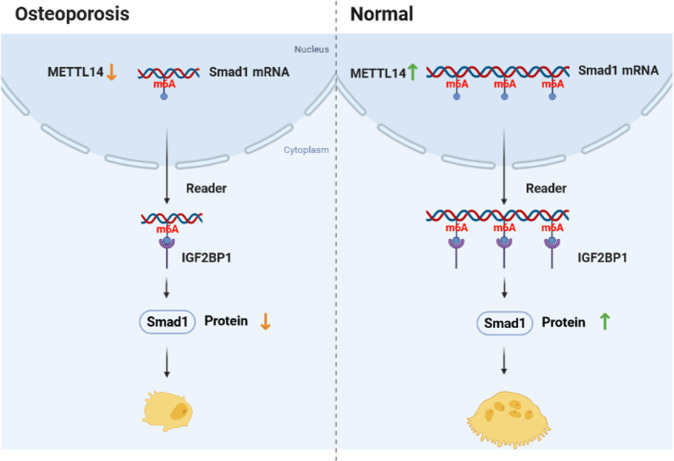
A graphic illustration shows the METTL14/SMAD1 axis in OP. In OP, METTL14 was downregulated and inhibited m6A methylation of SMAD1. The methylation suppression was regulated by m6A reader IGF2BP1, leading to the degradation of SMAD1 at mRNA and protein levels. In normal, METTL14 promoted m6A methylation of SMAD1 by modulating IGF2BP1, increasing SMAD1 protein expression.

In the present study, we found that the knockdown of METTL14 suppresses osteogenic differentiation by modulating m6A methylation of SMAD1, which was modulated by m6A reader IGF2BP1 (Fig. 9).

Bone development and remodeling require precise regulation of gene expression through epigenetics, including RNA modifications. M6A plays an important role in the development and metabolism of bone [13, 14]. It affects various bone diseases, such as OP, bone tumors, and arthritis [13, 15]. BMSCs maintain a balance between osteogenic and adipogenic differentiation to maintain bone health. Once the tendency from osteogenesis to adipogenesis, bone loss and changes in bone microstructure will occur, leading to OP and even fracture [16]. Growth evidence has indicated that m6A modification is associated with cell proliferation, differentiation, and apoptosis of BMSCs through its “writers”, “erasers”, and/or “readers” [17]. METTL3 is the most widely studied m6A “writer” in OP. Wu et al. reported that loss of METTL3 damages bone formation, inhibits the osteogenic differentiation potential of BMSCs, and increases bone marrow adiposity, whereas overexpression of METTl3 improves OP in estrogen-deficient mice [18]. Yan et al. revealed that silencing of METTL3 decreases m6A level, suppresses BMSCs differentiation, and reduces bone mass by m6A methylations of RUNX2 and miR-320 precursor [19]. METTL14 is an m6A “writer” second to METTL3, and also plays an important role in OP, but there is still relatively little research on it. Sun et al. found METTL14, a target of miR-103-3p, promoted m6A methylation and thereby promotes osteoblast activity, explaining the possible crucial role of METTL14 in OP [12]. However, further studies are needed on the effect of METTL14 on osteogenic differentiation in OP. Osteoporosis mice were established. The BV/TV, BMD, Tb.N, Tb.th, and BS were decreased, and Tb.Sp and fat cells were increased in the OVX mice. The data suggested that BMSCs tend to differentiate into adipoblasts rather than osteoblasts, which in turn leads to osteoporosis. Here, we found that m6A level was downregulated in patients with OP and OVX mice, but upregulated in osteogenic BSMCs, suggesting m6A methylation is involved in osteogenesis. Then, we explored which m6A “writer” regulates methylation modification. The results indicated that METTL14 and METTL3 were elevated in osteogenic BSMCs, but only METTL14 was downregulated in OP and OVX mice. Therefore, we choose METTL14 for research. The biological function results illustrated that knockdown of METTL14 suppressed osteogenesis and proliferation of BSMCs. Additionally, overexpression of METTL14 promoted the bone formation of OVX mice. Taken together, the findings suggested that METTL14 might be a target to improve OP.

To investigate the underlying mechanism, we found knockdown of METTL14 decreased the expression of SMAD1, SMAD5, and SMAD8, and only inhibited SMAD1 methylation. Therefore, SMAD1 has caught our attention. SMAD1 is crucial in bone morphogenetic protein (BMP) signaling transduction. SMAD1 could be phosphorylated and activated by the BMP receptor [20], which participates in regulating cell growth, differentiation, and death [21]. Moreover, SMAD1-mediated BMP signaling is necessary for both chondrogenesis and bone formation [22, 23]. BMP-SMAD signaling contributes to maintaining bone homeostasis, which defects may induce OP [24]. SMAD1 is associated with the osteogenic differentiation of several cell types, such as BSMCs. aortic valve interstitial cells, periodontal ligament stem cells, and adipose tissue-derived stem cells [25–28]. Importantly, SMAD1 is involved in OP by promoting the osteogenesis of BSMCs [25, 29]. Meanwhile, SMAD1 at phosphorylation can be detected in osteoclasts and has been proven to be vital in osteoclast differentiation and activity [30]. In the current study, SMAD1 was upregulated in osteogenic BMSCs, and downregulated in OVX mice and patients with OP. Transfection of SMAD1 vectors promoted the phosphorylation of SMAD1, and further played its function. SMAD1 reversed the inhibited effect of sh-METTL14 on osteogenesis and proliferation of BMSCs. Deletion of METTL14 suppressed the m6A level of SMAD1. The data suggested that METTL14 inhibited osteogenic differentiation of BSMCs by modulating m6A methylation of SMAD1. Moreover, we predicted multiple SMAD1 methylation sites and proved METTL14 only regulated SMAD1 m6A methylation at site 1 (GAACU). Silencing of METTL14 reduced the stability of SMAD1. Taken together, METTL14 bound to SMAD1 at the 1739-1743bp site, and depletion of METTL14 inhibited m6A methylation modification of SMAD1 by promoting SMAD1 degradation, leading to inhibition of BSMCs osteogenesis.

IGF2BP1, an RNA binding protein, also an m6A reader, participates in posttranscriptional regulation in cells. Targets of IGF2BP1 have higher stability and longer half-life [31]. Aberrant expression of IGF2BP1 is involved in pathological processes of diseases and regulates cellular processes, including growth, differentiation, and invasion [32]. A previous study has reported that lncRNA MALAT1 promotes osteogenesis by regulating IGF2BP1 [33]. Herein, we investigated whether IGF2BP1 is involved in downstream SMAD1 stabilization. The results showed that IGF2BP1 was positively related to SMAD1. IGF2BP1 reversed the decreased levels of SMAD1 induced by METTL14 knockdown. The findings suggested that the silencing of METTL14 inhibited SMAD1 m6A methylation by reducing IGF2BP1 expression.

In conclusion, our results indicated that METTL14 was downregulated in patients with OP and OVX mice. Knockdown of METTL14 inhibited osteogenic differentiation of BSMCs through positively regulating m6A methylation of SMAD1. We also found that METTL14 regulated m6A methylation of SMAD1 and thereby influenced the stability of SMAD1 at mRNA and protein levels. Moreover, overexpression of METTL14 increased the bone mass of OVX mice, suggesting METTL14 might improve postmenopausal OP. The findings suggested that METTL14 could be considered a novel therapeutic target for OP.

## Supplementary information

Point-by-point response

aj-checklist

Supplementary material

wb original data

## Data Availability

The datasets used and/or analyzed during the current study are available from the corresponding author on reasonable request.
