# Supplementary material for: Downregulation of METTL14 improves postmenopausal osteoporosis via IGF2BP1 dependent posttranscriptional silencing of SMAD1
Source: Cell Death Dis. 2022 Nov 1;13(11):919. doi: 10.1038/s41419-022-05362-y (PMC9626483; doi:10.1038/s41419-022-05362-y)
Supplement: Supplementary file 1 — Point-by-point response [file 41419_2022_5362_MOESM1_ESM.docx]

Dear editors and reviewers,

Thank you for your comments. We have substantially revised our manuscript after reading the comments provided by the reviewers. The point-by-point response has been listed in the following letter.

Reviewers’ Comments: 

Referee #2 (Remarks to the Author): 

This article clarified the effect of METTL14 on the osteogenesis of BSMCs and bone mass in mice after ovariectomy. Investigate METTL14-dependent m6A modification of SMAD1 during BMSCs osteoblastic differentiation.I have the following comments： 
1. Figure1d results is tissue extracted RNA. Which part of tissue did you extract RNA from, please explain in the Material and Methods. Figure 1e: the micro-CT image inverted? If not, please indicate that the trabecular bone in the sham group is more sparse. Figure1k: More fat cells were found in the bone marrow cavity of mice, please discuss the reason. The figure of HE cannot be proved, bone loss, the statement is incorrect (line 251) same as line 283.

Response: Thank you for your suggestion. Total RNA was extracted from mice right femurs in Fig. 1d. We supplemented the explain in the methods section. Please see line 150.

In Fig. 1e, we are sorry for the mistakes. We exchanged the images between the two groups to make sure they were accurate. Please see revised figure 1g.

In Fig. 1k, the results of H&E staining assay showed that the bone surface and bone volume of OVX mice was reduced, compared with the mice in the sham groups. We speculated that this is because BMSCs tend to differentiate into adipoblasts rather than osteoblasts. The images showed more fat cells proved our speculate. We have revised the description in the results and added the discussion. Please see revised figure 1h, line 258, and line 291.

2. Please carefully mark the days of differentiation, when did you get RNA after osteogenic differentiation induction? Please supply western blot analysis for the protein related osteogenic differentiation

Response: BMSCs were collected 21 days after differentiation and analyzed by ALP, ARS, and qRT-PCR. The differentiated times were added in the article. Please see line 106.

The expression of osteogenic markers was detected using western blot, and the results were added in the figures and article. Please see revised figure 1c, 1f, 3d, 4c, and 8d.

3. Discuss why there are differences in METTL3 and 14 expression between in vitro and in vivo data, and what is the METTL3 expression level in human samples? Why not the protein level, only the mRNA level in Figure 2?

Response: We first detected several methylated transferase expression in the differentiated and undifferentiated cells, and we found only METTL14 and METTL3 were differentially expressed. So, we then detected METTL14 and METTL3 levels in the sham and OVX mice, and found only METTL14 was differentially expressed. At last, METTL14 expression was detected in patients and control subjects, and the results showed that METTL14 was differentially expressed. Taken together, only METTL14 was differentially expressed in cells, mice, and human samples.

The aim of the experiments in Fig. 2 was to screen which gene plays a role in osteoporosis. Thus, we detected their mRNA levels. In general, differentially expressed genes also differ at the protein levels. Here, we supplemented their expression using western blot. Please see revised figure 2.

4. How are Figure 3g and 3h counted ? Please explain the methods in the material and methods

Response: The quantification of ALP and ARS staining assay was performed using the Image J software. We supplemented the methods in the article. Please see line 193 and 200.

5. In the Figure4 , positions of HE images are inconsistent and lack comparability.

Response: Thank you for your suggestion. Were-did the HE staining assay using the same sites of femurs. The results were revised figure 4e.

6. Why choose these genetic tests in figure5a, but not others? Please explain the reasons. The correlation of figure5f is relatively low, so it cannot be described as positively related, which is incorrect (Line 293).

Response: To explore the effects of METTL14 on signaling pathway, we selected several common signaling pathways to detect whether METTL14 dysregulation affected the abnormalities of these signaling pathways. These pathways are all important in the progression of osteoporosis.

In Fig. 5f, the results of correlation showed that r=0.7220 and p<0.0001. It is generally believed that P < 0.01 indicates a relationship between the two, while R > 0.7 indicates a very close relationship. Thus, we think that METTL14 and SMAD1 is positively related.

7. In Figure 6, why did Smad1 protein and mRNA degrade under NC? Please explain and discuss.

Response: There are a variety of RNA enzymes, proteases, lysosomes, and other substances in the cell, so the RNA and protein will be degraded with the increase of time. In this study, we focused on the effects of METTL14 on RNA and protein degradation. Knockdown of METTL14 induced the faster degradation of SMAD1.

8. The ALP and ARS staining of Figure8f and figure 3f are inconsistent. The overexpression of SMAD1with increase expression of pSmad1, please discuss.

Response: We are sorry we don’t quite understand your comments. We checked the results of Fig. 3f and 8f and confirmed that they were correct.

Overexpression of SMAD1 up-regulated its mRNA and protein levels. Since SMAD1 needs to activate its phosphorylation level to play its function, we detected its phosphorylation level and found that overexpression of SMAD1 could promote the phosphorylation of SMAD1. We have added the discussion in the article.

9. The expression and writing are incorrect, please check and revise the whole article.

Response: Thank you for your suggestion. The writing mistakes were revised.

Thank you again for your comments for our manuscript. We hope the revisions will be approved by you. We would like to hearing from you soon.

Yours sincerely,

Yuan Wang
